# Transcultural adaption and preliminary evaluation of “understanding low back pain” patient education booklet

**DOI:** 10.1186/s12913-019-4854-y

**Published:** 2019-12-30

**Authors:** Anna Sofia Simula, Hazel J. Jenkins, Riikka Holopainen, Petteri Oura, Katariina Korniloff, Arja Häkkinen, Esa-Pekka Takala, Mark J. Hancock, Jaro Karppinen

**Affiliations:** 1 Medical Research Center Oulu, Oulu University Hospital and University of Oulu, Oulu, Finland; 2 Faculty of Medicine, Center for Life Course Health Research, Faculty of Medicine, University of Oulu, P.O. Box 5000, 90014 Oulu, Finland; 3 Department of General Medicine Mikkeli Central Hospital (Essote), Mikkeli, Finland; 4 Department of Health Professions, Faculty of Medicine and Health Sciences, Macquarie University, Sydney, Balaclava Road, North Ryde, NSW 2109 Australia; 5 Department of Chiropractic, Faculty of Science and Engineering, Macquarie University, Sydney, Australia; 6 Faculty of Sport and Health Sciences, University of Jyväskylä, PO Box 35, FI-40014 Jyväskylä, Finland; 7 School of Health and Social Studies, JAMK University of Applied Sciences, PO BOX 207, FI-40101 Jyväskylä, Finland; 8 Department of Physical Medicine and Rehabilitation, Jyväskylä Central Hospital, Keskussairaalantie 19, 40620 Jyväskylä, Finland; 9 Finnish Institute of Occupational Health, P.O. Box 40, FI-00032 Helsinki and Oulu, Työterveyslaitos Finland

**Keywords:** Back pain, Patient education, Back pain imaging, Implementation, Primary care

## Abstract

**Background:**

Low back pain (LBP) is the number one cause of disability globally. LBP is a symptom associated with biological, psychological and social factors, and serious causes for pain are very rare. Unhelpful beliefs about LBP and inappropriate imaging are common. Practitioners report pressure from patients to provide inappropriate imaging. A recently developed patient education and management booklet, ‘Understanding low back pain’, was designed to target previously identified barriers for reducing inappropriate imaging. The booklet includes evidence-based information on LBP and supports communication between patients and practitioners. Our aim was to 1) describe the translation process into Finnish and 2) study patients’ and practitioners’ attitudes to the booklet and to evaluate if it improved patients’ understanding of LBP and practitioners’ ability to follow imaging guidelines.

**Methods:**

We translated the booklet from English to Finnish. Preliminary evaluation of the booklet was obtained from LBP patients (*n* = 136) and practitioners (*n* = 32) using web-based questionnaires. Open-ended questions were analysed using thematic analysis.

**Results:**

Approximately half of the patients reported that reading the booklet helped them to understand LBP, while a third thought it encouraged them to perform physical activity and decreased LBP-related fear. Eighty percent of practitioners reported that the booklet helped them to follow imaging guidelines. In addition, practitioners reported that they found the booklet helpful and that it decreased the need for imaging.

**Conclusions:**

The booklet seemed to be helpful in LBP management and in decreasing the need for LBP imaging according to patients and practitioners. Further research on the clinical effectiveness of the booklet in controlled study settings is needed.

**Trial registration:**

ISRCTN, ISRCTN14389368, Registered 4 April 2019 - Retrospectively registered; ISRCTN11875357, Registered 22 April 2019 - Retrospectively registered.

## Contributions to the literature

- The patient education booklet was designed to target previously identified barriers for reducing inappropriate imaging. The booklet includes evidence-based information on LBP and supports communication between patients and practitioners.
- This study evaluates patients’ and practitioners’ attitudes to the booklet and if it improved patients’ understanding of LBP and practitioners’ ability to follow imaging guidelines.
- The booklet seemed to be helpful in LBP management and in decreasing the need for LBP imaging.

## Background

Low back pain (LBP) is the most common cause of disability worldwide [1], causing suffering for individuals and financial burden for society in the form of increased health care costs and absence from work [2]. For most patients no serious or specific cause for pain can be demonstrated [3]. Many inaccurate or even harmful beliefs about LBP exist among patients and practitioners [4, 5]. Guidelines recommend avoidance of unnecessary imaging and support a biopsychosocial approach for LBP care [6, 7]. However, implementation of, and adherence to, guidelines is insufficient [8]. Therefore, there is a need for interventions, which help align care with international guidelines and address the common misconceptions about LBP.

Several educational booklets have been developed and partly tested since the 1990s [9, 10]. The use of patient education resources such as ‘The Back Book’ has been shown to have a positive effect on patients’ beliefs and clinical outcomes among primary care LBP patients [11]. Providing patients with information on managing mild LBP has shown to be cost-effective in an occupational health setting [12]. However, these educational resources only address patient knowledge and do not address the other identified barriers to appropriate LBP management or imaging. There is also strong evidence that most interventions have not been effective in reducing inappropriate imaging [13]. For example, education of practitioners and guideline dissemination do not seem to be effective strategies for reducing imaging for LBP [13].

Barriers to appropriate LBP management and to following imaging guidelines have been identified, including: patients’ and practitioners’ poor knowledge of appropriate LBP management; patients’ need for support and validation from the practitioner; and the practitioners’ use of imaging referral to help manage the consultation [13, 14]. Evidence-based patient education delivered by a medical practitioner during a consultation, including information about imaging guidelines, the nature of LBP, and appropriate management strategies, could help practitioners to support patients in their care, and facilitate better patient understanding of LBP and recovery.

An intervention aiming to address both patient and practitioner barriers, including appropriate imaging, has been previously developed [15]. The intervention included a patient education booklet designed to be used by practitioners during a LBP consultation to: 1) screen patients for possible serious pathology; 2) facilitate patient communication, education, and reassurance within the consultation including information about imaging issues; 3) provide a customised patient management plan; and 4) provide additional patient education information.

This booklet was translated into Finnish to be used in trials of LBP treatment. The aim of this study is to: 1) describe the translation process, and 2) study patients’ and practitioners’ attitudes to the booklet and to evaluate if it improved patients’ understanding of LBP and practitioners’ ability to follow imaging guidelines.

## Methods

**Fig. 1:**
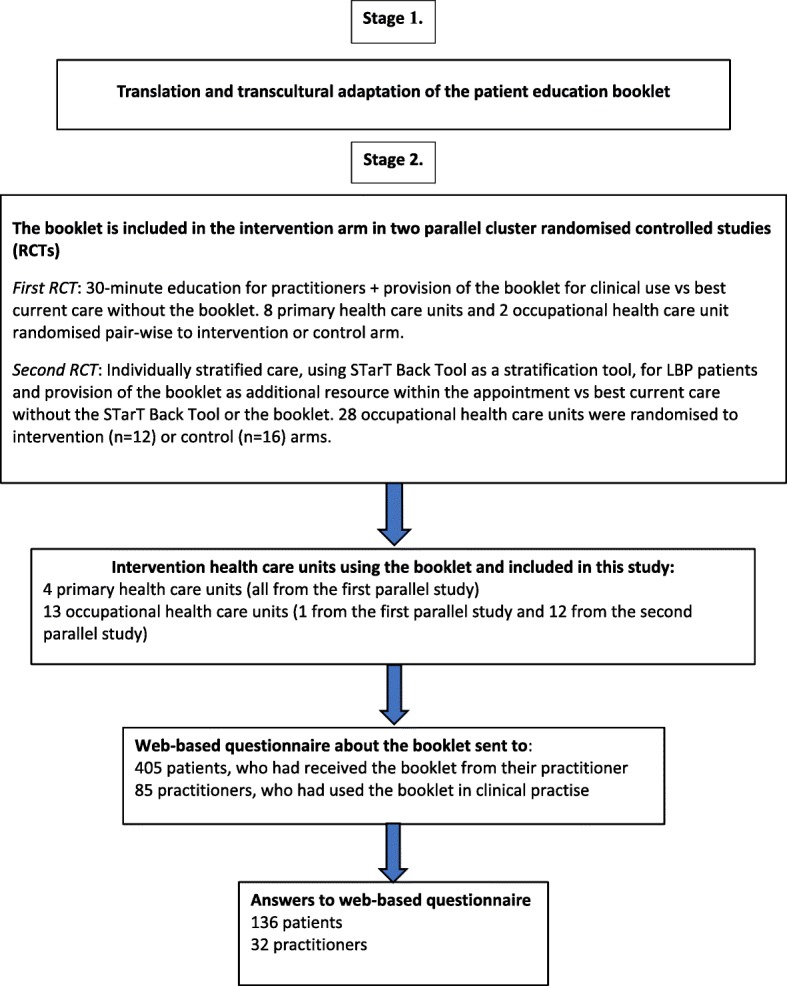
Flow chart of the study

This study involved two stages, as depicted in Fig. 1. Stage 1 involved the translation of the patient education booklet to Finnish using standardized procedures. Stage 2 was a sub-study of two parallel cluster randomized controlled trials were practitioners from 17 health care units used the translated booklet with patients in clinical practice as a component of the intervention. In this sub-study, a web-based questionnaire was used to evaluate the usefulness of the booklet from the perspective of both participating practitioners and LBP patients that the booklet was used with.

### Stage 1. Translation of the patient education booklet

The patient education booklet was developed in English by a team of researchers from Macquarie University, Sydney, Australia [15]. The development was based on the Behaviour Change Wheel [16] and preliminary testing was performed to assess the content of the patient education booklet, and its acceptability within clinical practice [15]. Experts in the field found the content of the booklet to be consistent with clinical guidelines, with appropriate language and format to facilitate practitioner use and patient understanding [15]. Practitioners thought the booklet would be helpful to decrease the use of non-indicated imaging. Health care consumers (lay people with a history of LBP) thought receiving the booklet from a practitioner would help reassure them about their LBP, and provide them with appropriate LBP management advice. The translation procedure of the booklet was performed according to the guidelines for transcultural adaptation of texts [17], with four iterations during the process.

#### Iteration 1

Two non-professional translators, whose native language was Finnish and with excellent skills in English, independently translated the original booklet from English to Finnish (KK, AH). Both translators were experienced professionals in rehabilitation medicine. After comparing the two translated versions along with the original English version, a consensus version was produced.

#### Iteration 2

A native English-speaking person with excellent skills in Finnish, who did not work in the field of medicine, performed a backward translation of the first iteration into English without being familiar with the original version beforehand. After comparing the content of the original and backward translated versions by all three translators, the observed differences were debated, and a second iteration was created.

#### Iteration 3

The first and second iterations of the booklet were presented to four external reviewers, who were experienced physicians of general health care (ASS), occupational health care (EPT), and rehabilitation (JK). The translators discussed the differences in translation with the reviewers until a consensus was reached.

#### Iteration 4 (final iteration)

The third iteration of the booklet was tested with ten LBP patients in an interview (ASS) after an appointment with a treating clinician. Duration of the interviews varied from 20 to 40 min. The patients were requested to report any difficulties in understanding the purpose or meaning of the words or the text. Each page was discussed separately. If the patients were unsure, they were asked to summarize the main message of the page. Based on this feedback minor modifications were made to the wording to optimise clarity and understanding. This version was then reviewed by a Finnish linguistic expert. The final version was checked by the same four external reviewers as during the third iteration.

### Stage 2. Evaluation of the booklet by LBP patients and practitioners

Evaluation of the booklet by LBP patients and practitioners was performed as part of two larger randomised controlled trials (RCTs), which utilised the booklet as part of the intervention. Practitioners and patients from primary health care units or occupational health care units in Finland that were allocated to the intervention group in the RCTs were invited to participate in this study. This included four primary health care units and one occupational health care unit from the first RCT, and 12 occupational health care units in the second RCT.

In the first RCT, eight primary health units and two occupational health care units in eastern Finland (in Mikkeli and Lappeenranta health care areas) during 2017–2018 were randomised pair-wise to intervention or control arms. Practitioners (physiotherapists and physicians) in the intervention arm attended a short 30 min education training session containing the main themes and theory of the booklet. The booklet was then provided to practitioners in the intervention arm to use as part of the care for LBP patients. In the second RCT, 28 occupational health care units across Finland during 2017–2018 were randomised to intervention (*n* = 12) or control (*n* = 16) arms. The intervention then consisted of individually stratified care, using the STarT Back Tool as a stratification tool, for LBP patients, and the booklet was provided to practitioners as an additional resource to use within the appointment. Practitioners also received comprehensive education in biopsychosocially oriented care for LBP in 4–7-day workshops. In both RCTs, it was requested that the practitioners in the intervention arms use the booklet with patients during the appointment, but they could choose to use it as they wanted. To evaluate the usefulness of the booklet, web-based questionnaires were sent to all participating practitioners in the intervention arms of both RCTs (four primary health care units and 13 occupational health care units in all) a few months after training and use of the booklet, and to all participating patients one to 2 weeks after consultation and recruitment. At the same time, patients were also sent a web-based questionnaire covering LBP characteristics and outcomes, within both parallel RCTs.

#### Practitioner and patient recruitment

All physicians and physiotherapists in the intervention arms were invited to participate in the study. In all, 85 practitioners consented to participate.

Patients were recruited by the participating practitioners. All patients from 18 to 65 years of age presenting to health care with LBP (with or without radicular pain) were included in the study. Exclusion criteria included suspicion of a serious cause for LBP, or LBP requiring urgent care. The first RCT was focusing on recurrent, subacute or chronic LBP. Patients were excluded if it was their first patient-reported contact to health care due to LBP and the episode duration was less than 2 weeks. The second RCT included acute to chronic patients with LBP.

#### Data collection

Patient demographics, characteristics of LBP, and previous imaging examinations as reported by patients were collected from the database of the RCTs. Frequency of LBP was enquired using the question ‘How often did you have LBP during the past 3 months?’ with answer options ‘less than half of the days’, ‘half of the days or more often’ and ‘every day’. Those having had pain at least half of the days were defined as ‘frequent LBP’. Intensity of LBP during the past week was defined on an 11-point numerical rating scale, where 0 = no pain and 10 = worst possible pain. Previous imaging examinations were assessed with the question: ‘Have you undergone medical imaging due to LBP during the past year?’.

Evaluation of the booklet was performed using a web-based questionnaire. Open and closed questions were used and analysed separately as described in the statistical methods section. Evaluation questions are shown in Tables 1 and 2. The questionnaire for patients consisted of 14 questions, which covered 4 main areas: 1) usability of the booklet; 2) influence on the desire for medical imaging; 3) usefulness in improving LBP understanding; and 4) usefulness in improving LBP outcomes. The questionnaire for practitioners consisted of 10 questions covering 3 main areas: 1) usability of the booklet; 2) barriers and facilitators to the appropriate use of imaging; 3) useful and useless elements of the booklet.

**Table 1:** Questionnaire to LBP patients for evaluation of the patient education booklet

Questionnaire for LBP patients	
Usability of the booklet	
1. Which factors facilitated your ability to read or pay attention to the information in the booklet during the appointment? (open answer)	
2. How could the booklet be further improved? (open answer)	
Influence on the desire for medical imaging	
3. Did you wish to undergo medical imaging due to LBP at the appointment? (Yes/no)	
4. Which factors affected your desire to undergo medical imaging due to LBP during the appointment? (open answer)	
5. Did the booklet have an influence on whether you wished or did not wish for imaging? (Yes/no)	
Usefulness in improving the understanding of LBP	
6. Which elements of the booklet did you find useful for understanding LBP? (open answer)	
Usefulness in improving the outcomes of LBP	
7. Which elements of the booklet did you find useful for the management of LBP? (open answer)	
8. Which elements of the booklet did you not find useful? (open answer)	
Likert scale questions, answer options: 1) disagree completely 2) disagree to some extent, 3) not agree or disagree, 4) agree to some extent, and 5) agree completely	
9. I believe that the booklet is useful for my understanding of LBP.	
10. After reading the booklet, I feel safer to be physically active.	
11. The booklet reduced my fears related to LBP.	
12. I believe that the booklet will enhance my recovery.	
13. The booklet is/was not useful for me at all.	
Additional question	
14. Did you receive the booklet from a physician or a physiotherapist?

**Table 2:** Questionnaire to practitioners for evaluation of the patient education booklet

Questions for practitioners	
Usability of the booklet	
1. Which factors facilitated or complicated the use of the booklet during the appointment? (open answer)	
2. How could the booklet be further improved? (open answer)	
Issues with medical imaging	
3. Which factors influenced (facilitated or complicated) the application of imaging guidelines during the appointment? (open answer)	
4. Did the booklet influence your initial imaging plan or was it easier to carry out because of the booklet? (open answer)	
Useful and un useful elements of the booklet	
5. Which elements of the booklet did you find particularly useful during the appointment? (open answer)	
6. Which elements of the booklet did you not find useful? (open answer)	
Likert scale questions, answer options: 1) disagree completely 2) disagree to some extent, 3) not agree or disagree, 4) agree to some extent, and 5) agree completely	
7. The booklet helps me to inform the patient about LBP.	
8. The booklet helps me to adhere to imaging guidelines.	
9. The booklet makes the appointment more laborious	
10. I believe that I will use the booklet in future appointments concerning LBP patients.	
11. The booklet aids in giving instructions to patients.	

#### Statistical methods

Analyses of closed questions and Likert scale questions were performed using descriptive statistics. In 5-point Likert scale questions three groups were formed: disagree (1 to 2), unsure (3), and agree (4 to 5). Among LBP patients, the associations between LBP frequency, intensity, and attitude towards the booklet (Likert scale questions) were analysed using the Chi-Square test. The distributions of responses were compared between the patients of frequent and non-frequent LBP, and between those with high and low intensity LBP. The analyses were carried out using SPSS version 25.

Open-ended questions were analysed using thematic analysis by one physician (ASS) and one physiotherapist (RH) [18]. Each open-ended question was analysed separately. All themes were derived from the data. Over several readings, close themes were combined. Rare reported comments that were not included in the main themes are not reported.

## Results

### Stage 1: Translation of the patient education booklet

#### Iterations 1 and 2

The Finnish version of the Patient education booklet was adapted using a process of forward-backward translation. Some semantic issues in the translation process were debated mainly concerning word choices to guarantee that meanings of the original content of the sentences were adequately captured in Finnish. There were some differences in the back-translated version compared to the original. Most of these were synonyms, but some words, for example ‘general fitness level’ and ‘safe exercises’, had a slightly different meaning in the first consensus version. All these discrepancies were solved by discussion, and the second Finnish consensus version was created.

#### Iteration 3

During the third iteration of the Finnish translation of the patient education booklet, the issues discussed by the external reviewers were related to readability and linguistic imprecisions. For example, in Finnish, the term ‘Low Back Pain’ is a long compound word with 12 letters (‘alaselkäkipu’) and therefore was translated to pain of lower back (8 and 4 letters; ‘alaselän kipu’) for easier readability. Some words and expressions were adopted from the Finnish Current Care Guidelines for LBP [7] for congruence. For example, the word ‘specific’ (‘spesifi’ in Finnish) is used in the Finnish LBP Current Care Guideline and was therefore used in the booklet even though the word has a foreign origin. ‘Irritation or compression of the nerves to the legs’ was translated as ‘radiating pain to the lower limbs or sciatica pain’, to be consistent with the Finnish guidelines and ensure understanding. The word ‘leg’ has two meanings in Finish: leg or foot, and was therefore translated as ‘lower limb’. To ensure understanding, we also wanted to use the common vernacular term ‘sciatica pain’ (‘iskiaskipu’ in Finnish). ‘Simple low back pain’ was further explained as ‘simple low back pain that does not require specific care’. The fourth red flag, ‘Severe pain which gets worse rather than better over several weeks’, was described without a time-related definition as ‘Severe pain which gets worse rather than better’.

#### Iteration 4

Six of the 10 LBP patients in the final iteration of the translation procedure were women; patients’ age range was 11 to 68 years, mean 31 years (missing data = 1); three were from secondary health care and seven from primary care; and duration of LBP ranged from a few days to 25 years. Three of the patients were students, two comprehensive schoolers, one on disability pension, one graduate Master of Arts, one unemployed chauffer, one pensioner, and one nurse. Six out of ten patients had no difficulties in understanding the purpose or meaning of the patient education booklet. Four patients pointed out some words and sentences, which needed to be defined more clearly in the final version of the booklet. For example, the widely used foreign word ‘spesifi’ (‘specific’), was explained as ‘tarkka’ (‘exact’ in English). The expression ‘to stay as active as possible’ was misunderstood by some patients as staying active in demanding examinations and treatment. Therefore, the expression was more accurately defined as staying active in daily life and physical activity. Of the red flags, the first item, ‘Difficulty passing or controlling urine or stool’, was misunderstood by some patients as a burning feeling when urinating, or constipation. Translation was therefore revised to ‘difficulties in emptying urine or stool or incontinence’. The website addresses for the online resources were considered too long and difficult and were shortened. Data saturation was achieved.

### Stage 2: Evaluation of the booklet by LBP patients and practitioners

#### Preliminary evaluation of the booklet by LBP patients

A total of 405 LBP patients received the booklet from their practitioner after providing informed consent to participate in the research. We received 136 responses to the questionnaire on the patient education booklet (34% response rate; shown in Fig. 1). Pain characteristics and imaging history of these patients were evaluated from the baseline self-reported web-based questionnaire related to the parallel studies. Half of the patients (*n* = 65, 51%, missing data = 8) reported frequent LBP, 41% (*n* = 53, missing data = 7) had high intensity of LBP, and previous imaging examinations had been performed for 32% (*n* = 41, missing data = 7).

**Fig. 2:**
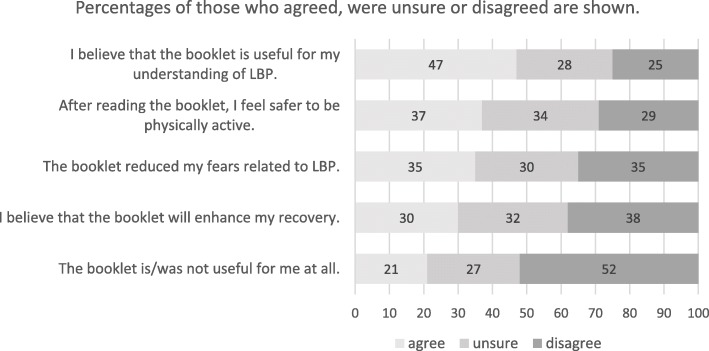
Evaluation of the patient education booklet by LBP patients (*n* = 136)

Based on Likert responses, 47% of the patients reported that reading the booklet helped them to understand LBP and was useful for them, while a third thought they recovered better, had less fear about their LBP, or were able to be more physically active (Fig. 2). Patients with more frequent LBP were more likely to have positive opinions about the booklet, while the intensity of LBP was not significantly associated with attitude towards the booklet (Table 3).

**Table 3:** Associations between patients’ opinion of the booklet and *low back pain characteristics*

Total *n* = 128	Agree % (n)	Unsure % (n)	Disagree % (n)	*p*-value
a) I believe that the booklet is useful for my understanding of LBP.				
All patients	46 (59)	29 (37)	25 (32)	
Frequent LBP^a^ Yes	57 (37)	25 (16)	18 (12)	0.040*
Frequent LBP^a^ No	35 (22)	33 (21)	32 (20)	
High intensity of LBP^b^ Yes	45 (24)	34 (18)	21 (11)	0.48
High intensity of LBP^b^ No	47 (35)	25 (19)	28 (21)	
b) I believe that the booklet will enhance my recovery.				
All patients	31 (39)	31 (40)	38 (49)	
Frequent LBP^a^ Yes	34 (22)	34 (22)	32 (21)	0.37
Frequent LBP^a^ No	27 (17)	28 (18)	44 (28)	
High intensity of LBP^b^ Yes	32 (17)	34 (18)	34 (18)	0.69
High intensity of LBP^b^ No	29 (22)	29 (22)	41 (31)	
c) The booklet reduced my fears related to LBP.				
All patients	34 (44)	31 (40)	34 (44)	
Frequent LBP^a^ Yes	45 (29)	28 (18)	28 (18)	0.043*
Frequent LBP^a^ No	24 (15)	35 (22)	41 (26)	
High intensity of LBP^b^ Yes	36(19)	32 (17)	32 (17)	0.90
High intensity of LBP^b^ No	33 (25)	31 (23)	36 (27)	
d) After reading the booklet, I feel safer to be physically active.				
All patients	37 (47)	35 (45)	28 (36)	
Frequent LBP^a^ Yes	42 (27)	38 (25)	20.0 (13)	0.11
Frequent LBP^a^ No	32 (20)	32 (20)	36 (23)	
High intensity of LBP^b^ Yes	36 (19)	40 (21)	24 (13)	0.62
High intensity of LBP^b^ No	37 (28)	32 (24)	31 (23)	
e) The booklet is/was not useful for me at all.				
All patients	22 (28)	27 (35)	51 (65)	
Frequent LBP^a^ Yes	14 (9)	25 (16)	62 (40)	0.027*
Frequent LBP^a^ No	30 (19)	30 (19)	40 (25)	
High intensity of LBP^b^ Yes	19 (10)	34 (18)	47 (25)	0.36
High intensity of LBP^b^ No	24 (18)	23 (17)	53 (40)	

Table 3 describes the association of pain characters and patients’ opinion for likert-scale questions related to the booklet. In all, 65 had frequent LBP and 53 had high intensity of LBP (missing pain data *n* = 8). *P*-values (*=significant) refer to the Chi-square test when comparing responses between patients with frequent (Yes vs. No) and high intensity (Yes vs. No) of LBP.

^a^Frequent LBP was defined as having pain half of days or more often during past 3 months

^b^ High intensity of LBP during past week was defined as 6 or more on 11 point-scale Numerical Rating Scale (NRS), where 0 = no pain and 10 = worst possible pain

In total, 42% of the patients reported wanting medical imaging because of LBP during the appointment. Of those, 21% reported that receiving the booklet decreased their desire for imaging compared to 9% of those who did not wish for imaging. Frequent LBP (*p* = 0.61), high intensity of LBP (*p* = 0.10) or previous imaging examinations (*p* = 0.22) were not associated with the desire for imaging.

Patients’ answers to the open-ended questions on the booklet were grouped into themes, shown in Table 4. The main themes, relating to how patients found the booklet useful to help their understanding of LBP, were: better understanding the reasons behind LBP; overall knowledge of LBP; knowledge of the commonness of LBP; clear and understandable information; knowledge that LBP is not serious; knowledge about the use of imaging for LBP; and the feeling of having better self-efficacy. Some patients reported that they were already familiar with the information in the booklet. The main themes, relating to how useful patients found the booklet for LBP management, were that the booklet provided: information about physical activity and exercise; advice in general; and self-care advice.

**Table 4:** Patient themes and direct quotes of specific elements within the booklet. n = 136

Open question	Theme	Direct quotes (patient code)	n
What elements of the booklet were useful for understanding LBP?			
	Explains for pain	*‘I Understand the reason of back pain, so it does not worry me that much anymore.’(242)*	17
	General knowledge of LBP	*‘The information from all aspects.’* (15)	16
	Knowledge on what is normal and typical	*‘Also, the explanation of pain and its commonness helps, because I know that I am not the only one suffering from that.’(214)*	9
	Clarity and understandability of the booklet	*‘Easy to read.’(84)*	9
	Understanding of pain not being dangerous	*‘That the feeling of pain isn’t dangerous.’* (9)	7
	Gives data on the role of imaging	*‘Principles of imaging.’(233)*	6
	Strengthening of self-efficacy	*‘By my own action, it is possible to have an influence on back healthiness.’* (19)	5
What elements of the booklet were useful for LBP management?			
	Encourages for exercise/physical activity	*‘Knowledge, that moving is remedy, so it is beneficial to move and seemingly it doesn’t matter if it increases the pain for a moment.’(126)*	36
	Advice in general	*‘Short, simple advice.’(34)*	25
	Self-care advice	*‘What I can do myself to improve my situation.’(240)*	13
Which factors facilitated your ability to read or pay attention to the information in the booklet during the appointment?			
	Support of practitioner	*‘The examinations made by the doctor and discussion as well as previous information I already have got.’(44)*	27
	Clarity and understandability of the booklet	*‘The chart was clear and the use of bold text drew attention to important topics.’(242)*	27
	Reading the booklet at home with time	*‘I got the booklet home with me.’* (7)	12
	Own interest/experience of LBP	*‘A brief booklet describing what lower back pain is and this is a topic that extensively interests me.’(81)*	9

Thematic analyzing method is used to create the themes from patients’ open answers for each question. There is one direct quote from each theme

Facilitators and barriers to reading or paying attention to the information within the booklet were also investigated. Only individual barriers were mentioned, and no clear themes related to barriers were found. Themes related to facilitators were: practitioner support; clarity of the booklet; and own interest in LBP.

Some patients provided suggestions for improving the booklet. In their open-ended answers, 25% of the patients reported a willingness to receive further information about LBP. Most frequently patients wanted extra information and advice about movements or exercise. Other information suggested to be included in the booklet was: advice on which practitioner or alternative practitioner could help them best; advice on mental health; and a clearer explanation of the reasons for back pain. For five patients, the booklet provoked negative feelings regarding imaging issues, or feelings of misunderstanding: *(*‘*Unnecessary banning of imaging’; ‘I feel, after reading the booklet that the defect is somewhere else but in my back. Patient’s pain suffering is underrated’).*

#### Preliminary evaluation of the booklet by practitioners

**Fig. 3:**
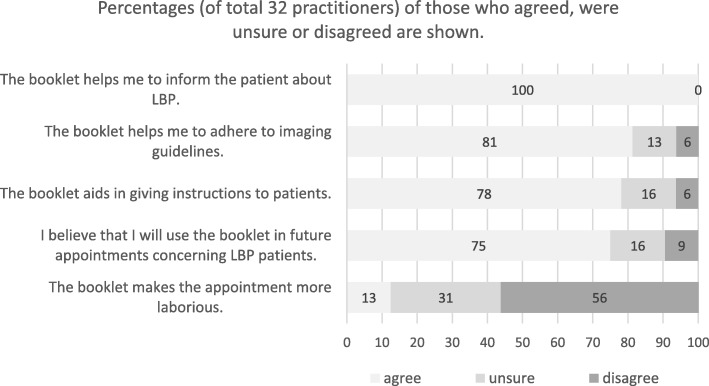
Evaluation of the patient education booklet by practitioners

In all, 32 practitioners out of 85 (response rate 38%, Fig. 1) answered the questionnaire; 10 were physicians and 22 were physiotherapists. Practitioners were located across Finland but mostly (38%) in the Mikkeli region. All practitioners thought that the booklet helped them inform the patient about LBP (Fig. 3). About 80% of the practitioners thought the booklet helped them to follow imaging guidelines and aided in giving instructions to patients, and 75% of the practitioners expressed their intention to use the booklet in the future (Fig. 3). Importantly, only 13% of practitioners thought that implementation of the booklet made the consultation more laborious.

Practitioners’ answers to open-ended questions about the booklet were grouped in themes. The main themes are shown in Table 5. The main facilitator was the usability of the booklet, practitioners found the booklet clear and easy to use. The main barriers to use were the lack of time, and difficulties in remembering to use the booklet. No specific themes for further improvement of the booklet arose from the practitioners’ answers.

**Table 5:** Practitioners themes and direct quotes of specific elements within the booklet (n = 32)

Open question	Theme	Direct quotes (GP = general practitioner, P=Physiotherapist).	n
Facilitators for the usability of the booklet			
	Good and clear content, easy to use	*‘A Clear and compact reference material supports the information given at a GP appointment’(GP7)*	12
Barriers for the usability of the booklet			
	Busy practice and remembering to use the booklet	*‘The Lack of time and haste hindered [the application of the booklet] substantially; if a suitable patient was present, then the booklet was not at hand…’(GP12)*	7
Which elements of the booklet did you find particularly useful during the appointment?			
	Comprehensive explanation of common issues	*‘The booklet in whole is practical, flow charts are clear, not too much information’(P19)*	14
	Imaging issues	*‘Indications for imaging and that asymptomatic people have similar findings’(P27)*	10
	Encourage to self-care	*‘ Encouragement towards self-care, endorsement of the general message.’(P28)*	5
Which elements of the booklet did you not find useful?			
	Blocks for management plan	*‘ Boxes in the management plan?’(P31)*	4
Facilitators influencing on following imaging guidelines			
	Good explanation	*‘That I have facts to tell patients about imaging and its necessity’(P26)*	8
	The Booklet	*‘The discussion at the GP appointment is supported by the general message of the booklet.’(P28)*	7
Barriers influencing on following imaging guidelines			
	Patient’s beliefs and wish for getting the reason for pain	*‘The patient has a strong belief, that imaging is needed’(GP5)*	19
Did the use of the booklet impact on imaging plan or did the booklet it easier to carry out the plan?			
	The booklet was helpful and decreased need for imaging	*‘Often the need of imaging decreased and the booklet supported realization of the rehabilitation plan’(P18)*	19
	The booklet did not have effect on imaging plan	*‘Hardly.’(P13)*	4

Thematic analyzing method is used to create the themes from practitioners’ open answers for each question. There is one direct quote from each theme

Specific elements of the booklet identified as useful by practitioners were the comprehensive explanation of common issues related to LBP and imaging issues*.* The most frequently reported barrier to following imaging guidelines was the patient’s beliefs and desire for imaging*.* Good explanation and using the booklet was reported to help deal with that conflict. The practitioners believed that using the booklet helped decrease the need for imaging.

## Discussion

The need to improve LBP management and adherence to appropriate LBP imaging guidelines is widely recognised [8, 19]. The aim of our study was to translate the new patient education booklet into Finnish and evaluate its usefulness from the perspectives of LBP patients and practitioners. The Finnish version of the booklet seems to be linguistically and culturally valid. Understanding was assured by one on one interviews with LBP patients with different social, health and age status. During the translation procedure, we made some changes to the wording of the booklet to improve its understandability among the Finnish population.

The primary aim of developing the booklet was to reduce inappropriate imaging among LBP patients. According to practitioners, the booklet helped them to follow imaging guidelines and reduce the need for imaging. The booklet seems to be helpful for LBP patients because it both modified beliefs, and encouraged and supported communication between patients and practitioners, which are the main basic elements of successful therapeutic alliance [20].

In our study, approximately half of the patients reported that reading the booklet helped them to understand LBP and it was useful for them, while one third thought they recovered better, had less fear about their LBP, or were able to be more physically active. We found that patients with frequent LBP, compared to those with less frequent pain, were more likely to report benefit from using the booklet. Patient education based on the biopsychosocial model seems to be an effective strategy for modifying beliefs about LBP, minimizing its consequences and increasing compliance to treatment [20]. There is moderate evidence that the addition of patient education to usual physiotherapy intervention improves disability in short-term among patients with chronic LBP [21].

Information about imaging for LBP in the booklet was not highlighted as important in our study from the patient’s point of view. Rather, they appreciated another explanation for their pain and encouragement for ongoing physical activity and self-care. Receiving information about imaging seemed to be more important for patients during the preliminary test of the English version of the booklet [15], which might be explained by the different populations, or methods of data collection. In our study, we used open written questions instead of interview questions which were focused more towards the imaging issues.

The most frequent barriers for practitioners to following imaging guidelines, according to our study, were ‘patients’ beliefs and desire for imaging’. The booklet seemed to be helpful by providing evidence-based and clear information, and therefore, useful in resolving some barriers to following imaging guidelines. In the open-ended questions, practitioners reported the booklet to be helpful and that it decreased the need for imaging. Similar expectations of practitioners were found in the preliminary testing of the English version of the booklet [15]. Patients reported that practitioner support was helpful for the successful use of the booklet. Clarity and understandability of the booklet were found useful according to both patients and practitioners. Previous research has reported that practitioners use imaging to help manage the consultation and most tested interventions did not decrease imaging rates [13, 22, 23]. Earlier studies on patients’ perspectives show that patients with LBP want clear explanations for their pain, written information and instructions, and support from the practitioner [24, 25]. The new patient education booklet combines biopsychosocial education with support for clinical decision making and patient communication including imaging issues [15].

Patients and clinicians were asked different questions about the usefulness of the booklet but overall the ratings were lower for patients than for clinicians. Despite this, approximately half of patients still considered the booklet as useful for understanding their LBP and only one fifth thought the booklet was not helpful at all (Fig. 2). Considering the primary aim of the booklet is to reduce inappropriate imaging among LBP patients (GP behaviour) and the booklet is a simple, cheap and low risk intervention, we think the 21% of patients not finding the booklet helpful is acceptable.

### Strengths and limitations of the study

A strength of the study was the fact that it was based on both quantitative and qualitative data. We addressed both patient and clinician perspectives in the assessment of the usability and usefulness of the booklet. Additionally, we were able to investigate if patients’ pain-related characteristics at presentation were associated with the results.

The moderate response rate (34% among patients and 38% among practitioners) may be considered a limitation of this study. We did not explore differences between the physiotherapists and physicians as we were not powered to do so and this is an area for future research. The tool was originally developed with physicians in Australia and our study provides preliminary data suggesting it is also considered useful by physiotherapists. This study did not collect data to evaluate whether the booklet actually changed imaging rates or patient outcomes. These will be evaluated in the future in our ongoing cluster randomised study.

## Conclusion

The Finnish version of the booklet seems to be linguistically and culturally valid. The new patient education booklet was reported to be helpful in LBP management according to both patients and practitioners. LBP patients reported increased understanding of LBP, increased motivation to perform physical activity and decreased LBP-related fear. Patients having frequent LBP seemed to be more likely to find the booklet useful. The booklet was helpful to facilitate practitioners’ adherence to imaging guidelines, by providing support for decision making and evidence-based resources to help the practitioner to manage the consultation and ensure the patient receives appropriate management. Further research of the clinical effectiveness and cost effectiveness of the booklet is needed.

## Data Availability

The datasets generated and analysed during the current study are not publicly available due to ongoing parallel studies but are available from the corresponding author on request.

## Abbreviations

LBP: Low back pain
